# Nutritional deficiencies after sleeve gastrectomy and Roux-en-Y gastric bypass at 10 years: secondary analysis of the SLEEVEPASS randomized clinical trial

**DOI:** 10.1093/bjs/znaf132

**Published:** 2025-07-04

**Authors:** Ilmari Saarinen, Marjatta Strandberg, Saija Hurme, Mika Helmiö, Sofia Grönroos, Anne Juuti, Risto Juusela, Pirjo Nuutila, Paulina Salminen

**Affiliations:** Department of Digestive Surgery, Division of Digestive Surgery and Urology, Turku University Hospital, Turku, Finland; Department of Surgery, University of Turku, Turku, Finland; Department of Surgery, Satasairaala Central Hospital, Pori, Finland; Emergency Care, Turku University Hospital, Turku, Finland; Department of Biostatistics, University of Turku and Turku University Hospital, Turku, Finland; Department of Digestive Surgery, Division of Digestive Surgery and Urology, Turku University Hospital, Turku, Finland; Department of Surgery, University of Turku, Turku, Finland; Department of Digestive Surgery, Division of Digestive Surgery and Urology, Turku University Hospital, Turku, Finland; Department of Surgery, University of Turku, Turku, Finland; Department of Surgery, Satasairaala Central Hospital, Pori, Finland; Department of Abdominal Surgery, Abdominal Centre, Helsinki University Hospital and University of Helsinki, Helsinki, Finland; Department of Surgery, Vaasa Central Hospital, Vaasa, Finland; Department of Endocrinology, Turku University Hospital, Turku, Finland; Turku PET Centre, University of Turku, Turku, Finland; Department of Digestive Surgery, Division of Digestive Surgery and Urology, Turku University Hospital, Turku, Finland; Department of Surgery, University of Turku, Turku, Finland

## Abstract

**Background:**

Long-term data on the prevalence of nutritional deficiencies after laparoscopic sleeve gastrectomy (LSG) and laparoscopic Roux-en-Y gastric bypass (LRYGB) in RCTs are lacking. The aim of this study was to compare nutritional deficiencies and adherence to vitamin supplements after LSG and LRYGB at 10 years.

**Methods:**

This was a predefined secondary analysis of the Finnish SLEEVEPASS (LSG *versus* LRYGB for severe obesity) multicentre RCT, with 10-year nutritional laboratory measurements and completed questionnaires on micronutrient supplement use, to assess the prevalence of micronutritional and macronutritional deficiencies and adherence.

**Results:**

Of 240 patients (121 LSG patients and 119 LRYGB patients), 228 were available for 10-year follow-up. Of these 228 patients, 190 (83.3%) were available for laboratory tests and 192 (84.2%) for questionnaires. There were no statistically significant differences between LSG and LRYGB in the prevalence of vitamin D insufficiency (10 of 94 (11%) *versus* 9 of 84 (11%) respectively; *P* = 0.545), the prevalence of hypocalcaemia (3 of 92 (3%) *versus* 1 of 83 (1%) respectively; *P* = 0.088), the prevalence of vitamin B12 deficiency (2 of 46 (5%) *versus* 0 of 45 (0%); *P* = 0.240), or mean vitamin B12 levels (*P* = 0.939). The prevalence of iron deficiency, defined by ferritin level, was statistically significantly lower after LSG compared with LRYGB (4 of 29 (14%) *versus* 12 of 29 (41%); *P* = 0.017), with a median ferritin level of 34 (interquartile range 20–54) µg/l after LSG and 20 (interquartile range 12–117) µg/l after LRYGB (*P* = 0.397). The LSG group had statistically significantly lower overall adherence to micronutritional supplements (70 of 99 (71%) *versus* 83 of 93 (89%) respectively; *P* = 0.002).

**Conclusion:**

Long-term micronutritional and macronutritional deficiencies were rare after both LSG and LRYGB, with similar deficiency rates. Only the prevalence of iron deficiency was more common after LRYGB compared with LSG. The overall adherence to micronutritional supplements was lower after LSG.

**Registration number:**

NCT00793143 (http://www.clinicaltrials.gov).

## Teaser

In this secondary analysis of the SLEEVEPASS RCT, nutritional deficiencies were relatively rare after laparoscopic sleeve gastrectomy and laparoscopic Roux-en-Y gastric bypass at 10 years. There was a difference between the procedures only in the prevalence of iron deficiency, which was lower after sleeve gastrectomy. The adherence to micronutrient supplements was also lower after sleeve gastrectomy.

## Introduction

The increasing global obesity epidemic is a threat to public health^1^. Metabolic bariatric surgery (MBS) is the most effective treatment of obesity, resulting in good and sustainable weight loss and remission of obesity-related diseases^2–9^. However, MBS is associated with postoperative micronutritional and macronutritional deficiencies, such as vitamin D, vitamin B12, and iron deficiencies, hypocalcaemia, and protein malnutrition^10–14^. Iron and vitamin B12 deficiencies can lead to anaemia, which is known to be a plausible complication of MBS^12–15^. To reduce the risk of deficiencies, MBS patients are recommended to use micronutrient supplements after surgery^11,16–19^. Poor adherence to supplements after MBS is common both at short-term and long-term follow-up^18–22^ and non-adherence seems to increase over time^18,20,23^.

Laparoscopic sleeve gastrectomy (LSG) and laparoscopic Roux-en-Y gastric bypass (LRYGB) are the two most common MBS procedures^24,25^, accounting for >80% of all MBS procedures performed globally^25^. Despite the popularity of LSG and LRYGB, long-term nutritional outcomes of RCTs are scarce^11,26^.

The aim of this predefined secondary analysis of an RCT (SLEEVEPASS)^5^ was to compare the prevalence of nutritional deficiencies, as well as the adherence to micronutrient supplements and the effect of supplements on the prevalence of deficiencies, after LSG and LRYGB at 10 years.

## Methods

The SLEEVEPASS study design and methods have been reported in detail previously^5,6^. Briefly, the SLEEVEPASS trial was a multicentre, multi-surgeon, open-label, randomized, clinical equivalence trial. The trial was conducted from March 2008 to June 2010 and a total of 240 patients were randomized to undergo either LSG or LRYGB. Criteria for eligibility were BMI >40 kg/m^2^ or >35 kg/m^2^ with an obesity-associated co-morbidity, age 18–60 years, and a previous failed adequate conservative treatment. Criteria for exclusion were BMI >60 kg/m^2^, significant eating or psychiatric disorder, active alcohol or substance abuse, active gastric ulcer disease, severe gastro-oesophageal reflux disease (GERD) with a large hiatal hernia, and previous bariatric surgery.

Postoperative micronutrient supplement recommendations included oral calcium-vitamin D, oral or intramuscular vitamin B12, and oral multivitamin supplements. Iron supplements were only prescribed for patients diagnosed with iron deficiency. At the time of study initiation, Nordic guidelines were not available and the use of supplements was based on multidisciplinary national guidance within the study group.

The trial was designed according to the principles of the Declaration of Helsinki and the study protocol was accepted by the ethics committees of all three participating hospitals in Finland (Turku, Vaasa, and Helsinki) and was registered at ClinicalTrials.gov (NCT00793143). Weight loss at 5 years, defined as percentage excess weight loss (%EWL), was the primary endpoint of the SLEEVEPASS trial^6^. %EWL was calculated as (preoperative weight − follow-up weight)/(preoperative weight − ideal weight for BMI 25) × 100%^27^.

The 10-year follow-up of the SLEEVEPASS study was completed in January 2021. For this predefined secondary analysis of the RCT, both nutritional laboratory values and responses to questionnaires on micronutrient supplement use were collected (see the *Supplementary material*). The laboratory tests included serum or plasma 25-hydroxyvitamin D, serum or plasma vitamin B12, blood haemoglobin, plasma ferritin, serum or plasma ionized calcium, plasma albumin, plasma magnesium, and plasma phosphate. Iron deficiency was defined by ferritin level as recommended by the WHO^28^.

The reference values and ranges used by local laboratories for vitamin D were as follows: deficiency, <25 nmol/l; insufficiency, 25–50 nmol/l; sufficiency, 51–74 nmol/l; and recommended reference range, 75–120 nmol/l. An ionized calcium level of 1.15–1.30 mmol/l was considered normal. The reference range for vitamin B12 was 145–570 pmol/l. Anaemia was defined according to national guidelines as low haemoglobin, with the following haemoglobin reference ranges: 117–155 g/l for women and 134–167 g/l for men. Iron status was measured using plasma ferritin, with reference ranges of 15–125 µg/l for women and 20–195 µg/l for men. The albumin reference range was 36–45 g/l, the magnesium reference range was 0.71–0.94 mmol/l, and the phosphate reference range was 0.76–1.41 mmol/l for women, 0.71–1.53 mmol/l for men aged 18–49 years, and 0.71–1.23 mmol/l for men aged ≥50 years.

### Statistical analyses

The main analyses were carried out for the modified intention-to-treat population (patients who never underwent surgery were excluded from this population). Additionally, the prevalences of nutritional deficiencies were also assessed using per-protocol analysis, where LSG patients who underwent conversion to LRYGB were analysed as LRYGB patients and conversions to other MBS procedures were excluded.

Continuous variables are presented as mean (s.d.) for normally distributed variables and as median (interquartile range (i.q.r.)) for non-normally distributed variables. Categorical variables are presented as frequencies and percentages, and differences between procedures for categorical variables and the association of anaemia with ferritin deficiency were evaluated using Fisher’s exact test. Differences between procedures for continuous ferritin values were evaluated using the non-parametric Mann–Whitney *U* test.

The difference between procedures regarding the prevalence of vitamin D insufficiency and the effect of calcium-vitamin D supplement use on the prevalence of vitamin D insufficiency were analysed using logistic regression analysis, including main effects of procedure and supplement use, and interaction of procedure and supplement use. In the analysis, the prevalence of insufficiency was compared with a joint prevalence of sufficiency and the recommended level. The final model only included main effects because interaction was not statistically significant. Results are presented as ORs, with 95% confidence intervals. The difference in mean vitamin B12 was evaluated using two-way ANOVA, with main effects of procedure and supplement use, and interaction of procedure and supplement use. If interaction was not statistically significant, the results are presented using main effects. Results of ANOVA are presented as least squares mean estimates, with 95% confidence intervals. The normality assumption was evaluated visually and using the Kolmogorov–Smirnov test. For vitamin B12, logarithmic transformation was used to achieve normality and back transformed least squares mean estimates are presented. Two-tailed *P* values <0.050 were considered statistically significant. Statistical analyses were performed using SAS software, version 9.4 for Windows (SAS Institute Inc., Cary, NC, USA).

## Results

**Fig. 1:**
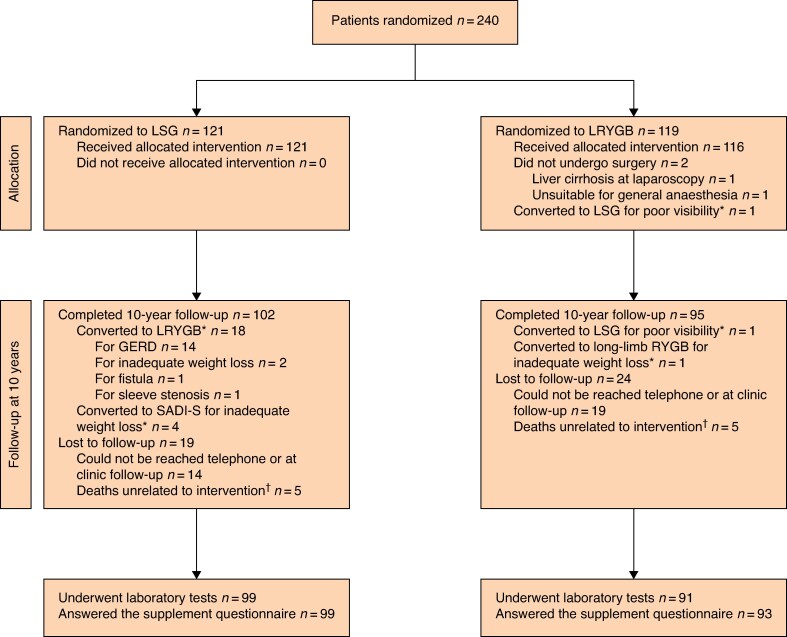
Flow of patients LSG, laparoscopic sleeve gastrectomy; LRYGB, laparoscopic Roux-en-Y gastric bypass; GERD, gastro-oesophageal reflux disease; SADI-S, single anastomosis duodenoileal bypass with sleeve gastrectomy; RYGB, Roux-en-Y gastric bypass. *Analysed according to intention to treat. †The specific causes of death were: one traffic accident, one drowning, one ketoacidosis; one pulmonary embolism, one uterine cancer, one cholangiocarcinoma, one lung cancer, one pancreatic cancer, and two alcohol overdoses.

The baseline characteristics of all 240 patients enrolled from 2008 to 2010 have been reported previously^6^. Two patients never underwent surgery during the 10-year follow-up. There were ten deaths unrelated to surgery. Out of the remaining 228 patients, 197 patients (86.4%) were available for 10-year follow-up and, out of these, 190 patients (83.3%) underwent laboratory tests and 192 patients (84.2%) were available for the supplement use information. The flow of patients is presented in *Fig. 1* and the numbers of available laboratory results at 10 years are presented in *Table 1*.

**Table 1:** Available nutrient values at 10 years

	All study patients		LSG		LRYGB		*P*
	n	Mean(s.d.) or median (i.q.r.)	*n*	Mean(s.d.) or median (i.q.r.)	*n*	Mean(s.d.) or median (i.q.r.)	
Vitamin D (nmol/l)	178	75 (22)	94	78 (24)	84	73 (20)	0.132*
Calcium (mmol/l)	175	1.23 (0.05)	92	1.24 (0.05)	83	1.23 (0.04)	0.095*
Vitamin B12 (pmol/l)	91	396 (271–513)	46	386 (282–471)	45	433 (253–543)	0.609†
Haemoglobin (g/l)	189	135 (14)	91	137 (15)	98	134 (13)	0.119*
Ferritin (µg/l)	58	28 (15–62)	29	34 (20–54)	29	20 (12–117)	0.397†
Magnesium (mmol/l)	178	0.8 (0.1)	93	0.8 (0.1)	85	0.9 (0.1)	0.176*
Phosphorus (mmol/l)	166	1.03 (0.15)	88	1.00 (0.14)	78	1.06 (0.15)	0.011*
Albumin (g/l)	184	38 (3)	97	38 (3)	87	38 (4)	0.930*

LSG, laparoscopic sleeve gastrectomy; LRYGB, laparoscopic Roux-en-Y gastric bypass; i.q.r., interquartile range. *Paired *t* test. ^†^Mann–Whitney *U* test.

The nutrient levels categorized by reference ranges and the prevalence of nutritional deficiencies are presented in *Table 2*. There was no statistically significant difference in the prevalence of vitamin D insufficiency between LSG and LRYGB at 10 years (10 of 94 (11%) *versus* 9 of 84 (11%) respectively; OR 0.73 (95% c.i. 0.27 to 2.01); *P* = 0.545). Patients using calcium-vitamin D supplements had statistically significantly less vitamin D insufficiency compared with non-adherent patients (8 of 121 (6.6%) *versus* 11 of 54 (20%) respectively; OR 0.26 (95% c.i. 0.09 to 0.71); *P* = 0.008). Adherence to nutritional supplements is presented in *Table 3*. The overall adherence to any supplement was statistically significantly lower after LSG (70 of 99 (71%) *versus* 83 of 93 (89%); *P* = 0.002). There was no statistically significant difference in the effect of supplement use on the prevalence of insufficiency between the procedures (operation × supplement use interaction *P* = 0.340). Vitamin D and calcium levels according to calcium-vitamin D adherence are presented in *Table 4*.

**Table 2:** Nutrient values categorized by reference ranges at 10 years

	All study patients	LSG	LRYGB	P*
**Vitamin D (nmol/l)**				
<25	0 of 178 (0.0)	0 of 94 (0)	0 of 84 (0)	0.413
25–50	19 of 178 (10.7)	10 of 94 (11)	9 of 84 (11)	
51–74	73 of 178 (41.0)	35 of 94 (37)	38 of 84 (45)	
75–120	80 of 178 (44.9)	44 of 94 (47)	36 of 84 (43)	
>120	6 of 178 (3.4)	5 of 94 (5)	1 of 84 (1)	
**Calcium** (**mmol/l)**				
<1.15	4 of 175 (2.3)	3 of 92 (3)	1 of 83 (1)	0.088
1.15–1.30	163 of 175 (93.1)	82 of 92 (89)	81 of 83 (98)	
>1.30	8 of 175 (4.6)	7 of 92 (8)	1 of 83 (1)	
**Vitamin B12 (pmol/l)**				
<145	2 of 91 (2)	2 of 46 (5)	0 of 45 (0)	0.240
145–570	71 of 91 (78)	37 of 46 (80)	34 of 45 (76)	
>570	18 of 91 (20)	7 of 46 (15)	11 of 45 (24)	
**Haemoglobin (g/l)**				
Under RL	30 of 189 (15.9)	15 of 98 (15)	15 of 91 (17)	0.783
Within RLs	155 of 189 (82.0)	80 of 98 (82)	75 of 91 (82)	
Over RL	4 of 189 (2.1)	3 of 98 (3)	1 of 91 (1)	
**Ferritin (µg/l)**				
Under RL	16 of 58 (28)	4 of 29 (14)	12 of 29 (41)	0.017
Within RLs	35 of 58 (60)	23 of 29 (79)	12 of 29 (41)	
Over RL	7 of 58 (12)	2 of 29 (7)	5 of 29 (17)	
**Albumin (g/l)**				
<36	47 of 184 (25.5)	25 of 97 (26)	22 of 87 (25)	>0.999
36–45	134 of 184 (72.8)	70 of 97 (72)	64 of 87 (74)	
>45	3 of 184 (1.6)	2 of 97 (2)	1 of 87 (1)	
**Magnesium (mmol/l)**				
<0.71	10 of 178 (5.6)	6 of 93 (7)	4 of 85 (5)	0.770
0.71–0.94	152 of 178 (85.4)	80 of 93 (86)	72 of 85 (84)	
>0.94	16 of 178 (9.0)	7 of 93 (8)	9 of 85 (11)	
**Phosphorus (mmol/l)**				
Under RL	4 of 166 (2.4)	3 of 88 (3)	1 of 78 (1)	0.606
Within RLs	159 of 166 (95.8)	84 of 88 (96)	75 of 78 (96)	
Over RL	3 of 166 (1.8)	1 of 88 (1)	2 of 78 (3)	

Values are *n* of *n* (%) unless otherwise indicated. LSG, laparoscopic sleeve gastrectomy; LRYGB, laparoscopic Roux-en-Y gastric bypass; RL, reference limit. *Fisher’s exact test.

**Table 3:** Nutritional supplement use

Supplement	All study patients	LSG	LRYGB	P*
Calcium-vitamin D	130 of 191 (68.1)	58 of 98 (59)	72 of 97 (77)	0.008
Vitamin B12	80 of 190 (42.1)	28 of 97 (29)	52 of 93 (56)	<0.001
Iron	17 of 189 (9.0)	4 of 96 (4)	13 of 93 (14)	0.022
Multivitamin	116 of 191 (60.7)	50 of 98 (51)	66 of 93(71)	0.005

Values are *n* of *n* (%) unless otherwise indicated. LSG, laparoscopic sleeve gastrectomy; LRYGB, laparoscopic Roux-en-Y gastric bypass. *Fisher’s exact test.

**Table 4:** Vitamin D and calcium levels according to calcium-vitamin D supplement adherence

	All study patients		LSG		LRYGB	
	Non-adherent	Adherent	Non-adherent	Adherent	Non-adherent	Adherent
**Vitamin D (nmol/l)**						
Mean (s.d.)	68.3 (24.0)	77.5 (19.9)	72.7 (25.8)	79.0 (20.7)	58.8 (16.3)	76.3 (19.4)
25–50	11 of 54 (20)	8 of 121 (7)	6 of 37 (16)	4 of 55 (7)	5 of 17 (29)	4 of 66 (6)
51–74	25 of 54 (46)	47 of 121 (39)	16 of 37 (43)	19 of 55 (34)	9 of 17 (53)	28 of 66 (42)
75–120	16 of 54 (30)	64 of 121 (53)	13 of 37 (35)	31 of 55 (56)	3 of 17 (18)	33 of 66 (50)
>120	2 of 54 (4)	2 of 121 (2)	2 of 37 (5)	1 of 55 (2)	0 of 17 (0)	1 of 66 (2)
**Calcium (mmol/l)**						
<1.15	1 of 50 (2)	2 of 121 (2)	1 of 34 (3)	1 of 56 (2)	0 of 16 (0)	1 of 65 (2)
1.15–1.30	46 of 50 (92)	115 of 121 (95)	30 of 34 (88)	51 of 56 (91)	16 of 16 (100)	64 of 65 (99)
>1.30	3 of 50 (6)	4 of 121 (3)	3 of 34 (9)	4 of 56 (7)	0 of 16 (0)	0 of 65 (0)

Values are *n* of *n* (%) unless otherwise indicated. LSG, laparoscopic sleeve gastrectomy; LRYGB, laparoscopic Roux-en-Y gastric bypass.

The mean estimate of vitamin B12 was 372 (95% c.i. 302 to 459) pmol/l after LSG and 379 (95% c.i. 318 to 444) pmol/l after LRYGB (*P* = 0.939). There was no difference in the mean estimate of vitamin B12 for patients using vitamin B12 supplements compared with patients not using vitamin B12 supplements (398 (95% c.i. 322 to 493) pmol/l *versus* 351 (95% c.i. 299 to 412) pmol/l respectively; *P* = 0.348). Between LSG and LRYGB, there was no statistically significant difference in the effect of supplement use (operation × supplement use interaction *P* = 0.120). Vitamin B12 levels according to vitamin B12 supplement adherence are presented in *Table 5*.

**Table 5:** Vitamin B12 levels according to vitamin B12 supplement adherence

	All study patients		LSG		LRYGB	
	Non-adherent	Adherent	Non-adherent	Adherent	Non-adherent	Adherent
**Vitamin B12 (pmol/l)**						
Median (i.q.r.)	365 (271–459)	472 (273–667)	389 (298–1326)	377 (243510)	303 (227–429)	505 (273–669)
<145	1 of 51 (2)	1 of 37 (3)	1 of 35 (3)	1 of 8 (13)	0 of 16 (0)	0 of 29 (0)
145–570	44 of 51 (86)	24 of 37 (65)	29 of 35 (83)	5 of 8 (63)	15 of 16 (94)	19 of 29 (66)
>570	6 of 51 (12)	12 of 37 (32)	5 of 35 (14)	2 of 8 (25)	1 of 16 (6)	10 of 29 (35)

Values are *n* of *n* (%) unless otherwise indicated. LSG, laparoscopic sleeve gastrectomy; LRYGB, laparoscopic Roux-en-Y gastric bypass; i.q.r., interquartile range.

After LSG, there was a statistically significantly lower prevalence of iron deficiency, defined by ferritin level, compared with LRYGB (4 of 29 (14%) *versus* 12 of 29 (41%) respectively; *P* = 0.017) (*Table 2*). The per-protocol analysis results (*Table S1*) were similar to the intention-to-treat analysis results, except for iron deficiency (3 of 20 (15%) for LSG *versus* 12 of 37 (32%) for LRYGB; *P* = 0.283). The median ferritin level was 34 (i.q.r. 20–54)  µg/l in the LSG group and 20 (i.q.r. 12–117) µg/l in the LRYGB group (*P* = 0.397) (*Table 1*). The prevalence of anaemia, defined by haemoglobin level, did not differ between the procedures (15 of 98 (15%) after LSG *versus* 15 of 91 (17%) after LRYGB; *P* = 0.783) (*Table 2*). There was no statistically significant association between ferritin deficiency and anaemia (*P* = 0.283). The mean calcium, albumin, magnesium, and phosphorus levels are described in detail in *Table 1*.

## Discussion

In this predefined 10-year secondary analysis of the SLEEVEPASS trial, long-term micronutritional and macronutritional deficiencies were quite rare after both LSG and LRYGB, with similar deficiency rates for vitamin D, calcium, and vitamin B12. Iron deficiency, defined by ferritin level, was common and statistically significantly higher after LRYGB compared with LSG, but ferritin deficiency was not associated with anaemia. The adherence to nutritional supplements was lower after LSG compared with LRYGB. Despite the RCT nature of the follow-up and the high follow-up rate, the overall adherence to supplements was quite low.

In this study, the prevalence of vitamin D insufficiency was lower (11%) than the 42% reported in a recent meta-analysis of studies on LRYGB, which reported major heterogeneity in the studies and definitions of vitamin D deficiency^29^. The results of the present study are also in contrast with another recent meta-analysis reporting vitamin D deficiency to be less frequent after LSG^11^. However, this meta-analysis only included short- to mid-term follow-up studies and only 2 of 24 of the included studies were RCTs^11^. A retrospective study found the prevalence of hypocalcaemia after MBS to be 3.6%^30^, which is in line with the present study. Shah *et al*.^30^ reported a prevalence of 9.3% after LSG and 1.9% after LRYGB, but presumed this to be due to selection bias, as patients with renal insufficiency tended to undergo LSG and were thus over-represented in the LSG group.

The prevalence of vitamin B12 deficiency (2%) in this study is in line with a previous report showing a prevalence of 2.0% 20 years after LRYGB^14^. In a recent meta-analysis of LSG studies, the prevalence of vitamin B12 deficiency remained unchanged (4%) from baseline to 5 years^15^. Regarding the difference between LSG and LRYGB, the results of the present study are in contrast with the recent meta-analysis of ten RCTs, where patients undergoing LRYGB were reported to have an increased risk of vitamin B12 deficiency^26^. This meta-analysis consisted mostly of short-term follow-up RCTs^26^ and there is evidence that the prevalence of vitamin B12 deficiency peaks at 2–3 years after surgery before decreasing again^13,14^. It may be possible that the vitamin B12 level fluctuates more after LRYGB than LSG due to larger anatomical and physiological alterations.

The prevalence of anaemia, defined by haemoglobin level, in this study was in line with previous long-term results^14,15^. Also in agreement with the results of the present study, the recent meta-analysis showed no difference in anaemia between LSG and LRYGB^26^. Previous research on iron deficiency after MBS is inconsistent due to varying markers and reference ranges. Ferritin is currently the recommended marker for iron deficiency^28^, although it has its limitations, as it might increase due to an inflammatory response^31^. The prevalence of low ferritin after LSG has been reported to be 27% at 5 years^15^, which is substantially higher than in this study (14%). The prevalence of low ferritin in the LRYGB group in this study was considerably higher (41%) than the prevalence of iron deficiency in many previous studies, but long-term results are scarce^12–14,32,33^. The results of the present study are in agreement with the 10-year results of a retrospective study showing a high prevalence of low ferritin (37.5%) after LRYGB^34^. The recent meta-analysis found no difference in iron deficiency between LSG and LRYGB^26^, in contrast with the results of the present study. The results of the present study did not show an association between ferritin deficiency and anaemia, although previous research indicates that anaemia after MBS seems to be caused by iron deficiency^14,15^. The findings on ferritin level in this study may be affected by the small sample size.

Iron supplements were only prescribed for patients diagnosed with iron deficiency. Iron supplements were used less frequently in the LSG group than in the LRYGB group, supporting the finding that deficiency was more prevalent after LRYGB. The results of the present study indicate that the risk of iron deficiency at long-term follow-up should be taken into account in optimal procedure selection, as this may be of particular importance in treating patients with other risk factors for iron deficiency, for example women of fertile age. To some extent, patients who have undergone LRYGB, may require closer monitoring for iron deficiency^35–38^. The main absorption site of iron is the duodenum, which could explain the high prevalence of iron deficiency in the LRYGB group^38^.

The long-term follow-up is probably the most important reason for low adherence in the present study, as the comparison studies are mostly short-term follow-up studies^18,20,21^. This result is highlighted by the fact that self-reported data are known to demonstrate overoptimistic adherence^39,40^. The present study showed that patients who underwent LSG had lower adherence to all supplements. Previous research on this difference between the procedures is inconclusive^18,22^. Patient-reported reasons for poor adherence include forgetfulness, lack of consistent habits, gastrointestinal side effects, unpleasant taste or smell, and costs^22,23,41^. Younger age, experience of side effects, mental health problems, and lack of regular medication before surgery have been identified as objective risk factors for poor adherence^18,42^. MBS patients have been shown to experience trouble taking their supplements^41^, which may be enhanced by negative attitudes towards supplements and dissatisfaction with the instructions and lack of tailored supplements^22^. A decrease in adherence over time has been reported in short-term studies^18,20,23^ and is also likely seen in this study, despite the RCT study design and follow-up rate.

The adherence to calcium-vitamin D supplements in this study is in line with a recent long-term study on adherence in LRYGB patients^19^. Adherent patients had higher vitamin D levels, which is in line with previous studies^11,19,29,43,44^. The adherence to vitamin B12 supplements in this study (42%) was considerably lower compared with some reported very high adherence rates, of up to 95%^19^. In contrast with the results of the present study, with vitamin B12 supplements having no effect on vitamin levels, Bjerkan *et al*.^19^ also found that adherent patients had higher vitamin B12 levels. The supplement questionnaire used in this study did not differentiate between oral and intramuscular vitamin B12 supplements, but it is presumable that many patients used intramuscular supplements, which are not affected by alterations to the gastrointestinal tract.

This study is limited by small sample sizes, especially for ferritin level and iron deficiency. Also, the number of preoperative values is low, despite being a predefined secondary outcome, and this prevents comparison between baseline and 10 years. Vitamin B12 was measured as serum or plasma vitamin B12, which is not currently the recommended marker for vitamin B12 deficiency; however, serum or plasma vitamin B12 was also used in all reference studies, facilitating comparison of the results. Another limitation is that sufficient information on folate values is lacking. Folate was measured as red blood cell folate, which is not currently recommended as a marker for folate deficiency. In addition, there were some laboratory technical issues during the follow-up visits, confusing the folate results, and, consequently, folate values could not be analysed. A major limitation is that only self-reported data were used for assessing adherence to supplements. More precise results could be achieved by combining data from questionnaires with objective data from prescription or reimbursement records^39^.

Despite these limitations of the study, long-term micronutritional and macronutritional deficiencies were quite rare for vitamin D, vitamin B12, and calcium after both LSG and LRYGB, with similar deficiency rates. The overall adherence to micronutritional supplements was lower after LSG. The prevalence of iron deficiency, defined by ferritin level, was common and statistically significantly higher after LRYGB compared with LSG, which needs to be taken into consideration in both procedure selection and long-term follow-up.

## Supplementary Material

znaf132_Supplementary_Data

## Data Availability

The data will be made available from the corresponding author upon reasonable request.
