# Supplementary material for: Nutritional deficiencies after sleeve gastrectomy and Roux-en-Y gastric bypass at 10 years: secondary analysis of the SLEEVEPASS randomized clinical trial
Source: Br J Surg. 2025 Jul 4;112(7):znaf132. doi: 10.1093/bjs/znaf132 (PMC12231607; doi:10.1093/bjs/znaf132)
Supplement: znaf132_Supplementary_Data [file znaf132_supplementary_data.zip › Supplementary_material.docx]

**Nutritional deficiencies after sleeve gastrectomy and Roux-en-Y gastric bypass at 10 years: A secondary analysis of the SLEEVEPASS randomized clinical trial**

Ilmari Saarinen, MD^1,2,3^; Marjatta Strandberg, MD, PhD ^4^; Saija Hurme, MSc ^5^; Mika Helmiö, MD, PhD ^1,2^; Sofia Grönroos, MD, PhD ^1,2,3^; Anne Juuti, MD, PhD^6^; Risto Juusela, MD^7^; Pirjo Nuutila, MD, PhD^8,9^; Paulina Salminen MD, PhD^1,2.^

^1^ Department of Digestive Surgery, Division of Digestive Surgery and Urology, Turku University Hospital, Turku, Finland

^2^ Department of Surgery, University of Turku, Turku, Finland

^3^ Department of Surgery, Satasairaala Central Hospital, Pori, Finland

^4^ Emergency Care, Turku University Hospital, Turku, Finland

^5^ Department of Biostatistics, University of Turku and Turku University Hospital, Turku, Finland

^6^Department of Abdominal Surgery, Abdominal Centre, Helsinki University Hospital and University of Helsinki, Helsinki, Finland

^7^Department of Surgery, Vaasa Central Hospital, Vaasa, Finland

^8^Department of Endocrinology, Turku University Hospital, Turku, Finland

^9^Turku PET Centre, University of Turku, Turku, Finland

Corresponding author:

Paulina Salminen, MD, PhD, FACS (Hon)

paulina.salminen@tyks.fi

University of Turku, Department of Surgery

Turku University Hospital, Division of Digestive Surgery and Urology

P.O. Box 52

20521 Turku, Finland

| **Supplementary Materials** |  |
| --- | --- |
| **Supplementary Methods**  Supplement questionnaire  **Supplementary Results** | *pag. 2* |
| Supplementary table 1: Per-protocol nutrient values categorized by reference ranges at 10 years | *pag. 3* |
|  |  |

|  | **All study patients** | **LSG** | **LRYGB** | **p value*** |
| --- | --- | --- | --- | --- |
| **Vitamin D (nmol/l)** |  |  |  |  |
| <25 | 0/175 (0.0) | 0/79 (0.0) | 0/96 (0.0) | 0.139 |
| 25-50 | 19/175 (10.9) | 8/79 (10.1) | 11/96 (11.5) |  |
| 51-74 | 71/175 (40.6) | 27/79 (34.2) | 44/96 (45.8) |  |
| 75-120 | 79/175 (45.1) | 39/79 (46.4) | 40/96 (41.7) |  |
| >120 | 6/175 (3.4) | 5/79 (6.3) | 1/96 (1.0) |  |
| **Calcium** (**mmol/l)** |  |  |  |  |
| <1.15 | 4/172 (2.3) | 3/76 (4.0) | 1/96 (1.0) | 0.415 |
| 1.15-1.30 | 160/172 (93.0) | 69/76 (90.8) | 91/96 (94.8) |  |
| >1.3 | 8/172 (4.7) | 4/76 (5.3) | 4/96 (4.2) |  |
| **Vitamin B12 (pmol/l)** |  |  |  |  |
| <145 | 2/90 (2.2) | 1/44 (2.3) | 1/46 (2.2) | 0.713 |
| 145-570 | 70/90 (78.0) | 36/44 (81.8) | 34/46 (73.9) |  |
| >570 | 18/90 (20.0) | 7/44 (15.9) | 11/46 (23.9) |  |
| **Hemoglobin** **(g/l)** |  |  |  |  |
| Under RL | 29/186 (15.6) | 13/82 (15.9) | 16/104 (15.4) | 0.783 |
| Within RLs | 153/186 (82.3) | 66/82 (80.5) | 87/104 (83.7) |  |
| Over RL | 4/186 (2.2) | 3/82 (3.7) | 1/104 (1.0) |  |
| **Ferritin (µg/l)** |  |  |  |  |
| Under RL | 15/57 (27.6) | 3/20 (15.0) | 12/37 (32.4) | 0.283 |
| Within RLs | 35/57 (60.3) | 15/20 (75.0) | 20/37 (54.1) |  |
| Over RL | 7/57 (12.1) | 2/20 (10.0) | 5/37 (13.5) |  |
| **Albumin (g/l)** |  |  |  |  |
| <36 | 47/181 (26.0) | 23/81 (28.4) | 24/100 (24.0) | 0.582 |
| 36-45 | 131/181 (72.4) | 56/81 (69.1) | 75/100 (75.0) |  |
| >45 | 3/181 (1.7) | 2/81 (2.5) | 1/100 (1.0) |  |
| **Magnesium** **(mmol/l)** |  |  |  |  |
| <0.71 | 10/175 (5.7) | 6/77 (7.8) | 4/98 (4.1) | 0.375 |
| 0.71-0.94 | 149/175 (85.1) | 66/77 (85.7) | 83/98 (84.7) |  |
| >0.94 | 16/175 (9.1) | 5/77 (6.5) | 11/98 (11.2) |  |
| **Phosphorus,**  **(mmol/l)** |  |  |  |  |
| Under RL | 4/163 (2.5) | 2/73 (2.7) | 2/90 (2.2) | >0.999 |
| Within RLs | 156/163 (95.7) | 70/73 (95.9) | 86/90 (95.6) |  |
| Over RL | 3/163 (1.8) | 1/73 (1.4) | 1/90 (2.2) |  |

Abbreviations: LSG, laparoscopic sleeve gastrectomy; LRYGB, laparoscopic Roux-en-Y gastric bypass; RL, reference limit

*Fisher’s exact test

The values are n (%) unless otherwise indicated.

**Per-protocol analysis: nutrient values categorized by reference ranges at 10 years**
