# Supplementary material for: Nutritional deficiencies after sleeve gastrectomy and Roux-en-Y gastric bypass at 10 years: secondary analysis of the SLEEVEPASS randomized clinical trial
Source: Br J Surg. 2025 Jul 4;112(7):znaf132. doi: 10.1093/bjs/znaf132 (PMC12231607; doi:10.1093/bjs/znaf132)
Supplement: znaf132_Supplementary_Data [file znaf132_supplementary_data.zip › Trial Protocol_SLEEVEPASS English translation 10-y follow-up ethical amendment 4.2.2018.docx]

Laparoscopic Gastric Bypass versus Sleeve Gastrectomy to Treat Morbid Obesity (SLEEVEPASS)

Trial protocol version 4.2.2018 (original 19.2.2008)

Paulina Salminen, MD, PhD

A prospective randomized multicenter study: laparoscopic gastric bypass versus laparoscopic sleeve gastrectomy in the treatment of morbid obesity

**Purpose of the study**

In recent years morbid obesity has been recognized a serious worldwide health crisis. In the US obesity in considered the second most common cause of premature death after smoking. It has been estimated that 400 000 deaths per year are related to obesity and its complications ^(13)^. Body mass index (BMI) of Finnish people has been increasing and obesity has become more common during the last two decades. In 2002 the mean BMI was 27,0 kg/m² among working age males and 25,9 kg/m² among females in Finland. 66 % of the males and 49 % of the females had BMI ≥ 25 kg/m² and every fifth had BMI ≥ 30 kg/m² ^(10)^.

Obesity is related to increased morbidity and mortality. The increased mortality in mainly caused by obesity related comorbidities such as insulin resistance, type 2 diabetes, hypertension and dyslipidemia. ^(13)^ In addition to this, obesity is related to many other diseases such as metabolic syndrome, coronary artery disease, stroke, obstructive sleep apnea, gout, gall stones, fatty liver disease, arthritis, asthma and certain cancers (postmenopausal breast, uterus, colon and kidney cancer ^(2)^.

Bariatric surgery is considered superior to conservative treatment regarding weight loss and resolution of comorbidities in patients with BMI ≥ 40 kg/m² ^(12)^. In two recent studies bariatric surgery decreased common morbidity compared to conservative treatment ^(3, 17)^. Death by heart disease, diabetes and cancer was less common after gastric bypass operation compared to control group ^(3)^.

The most common operative technique worldwide is gastric bypass (65 % of all procedures) and over half of these are performed by laparoscopy ^(6)^. In this operation a small 30 ml gastric pouch is created, the biliopancreatic limb is measured (approximately 50 cm) by graspers and an antecolic end-to-side gastrojejunostomy is created (circular or linear staplers). The alimentary limb is measured by graspers at 150 cm and a side-to-side jejuno-jejunostomy is created.

The weight loss is based on limited food intake by the small gastric pouch, partial malnutrition by the ileal bypass and possible hormonal changes. ^(12)^ Mean excessive weight loss (%) after gastric bypass is 25 % at ten-year follow-up. In the same SOS study at ten-year follow-up regarding conservative treatment equaled 1,6 % excessive weight gain. Surgery was also superior to conservative treatment considering diabetes, hypertriglyseremia, low high density lipoprotein, hypertension and hyperuricaemia. ^(17)^ Mortality after gastric bypass is 0,5 – 2,5 % and morbidity is 10 – 20 % ^(4, 12)^.

Laparoscopic sleeve gastrectomy is a relatively new operative technique. It was originally intended as a bridge procedure for high-risk super obese patients (BMI > 60) preceding the definitive bariatric procedure ^(15)^. Sleeve gastrectomy is created narrow along a 33-35 Fr calibration bougie using linear staplers preserving the majority of the antrum. The weight loss is based on limited food intake by the narrow gastric tube created in the procedure and decrease of ghrelin hormone secreted from the resected fundus of the stomach. In the last few years sleeve gastrectomy has been used as a single procedure for patients with BMI 35 – 60. The initial promising results in terms of weight loss and the resolution of comorbidities have been comparable to gastric bypass at short-term follow-up. However, long-term results of sleeve gastrectomy are very scarce ^(9, 14)^.

Morbid obesity by itself increases risk for venous thrombosis and pulmonary embolism. After bariatric surgery, possible serious complications are hemorrhage, anastomotic or staple line leakage and related infection and stricture of the anastomosis. Some of these complications must be treated by reoperation. After laparoscopic gastric bypass the risk for serious complication is 0.9–5.1 % ^(7, 8, 16)^ and mortality is 0,16 % ^(5)^. The risk for serious complication after laparoscopic sleeve gastrectomy is 2,9 % ^(11)^, but studies regarding mortality and long-term results are lacking.

**Aim of the study**

The aim of the study is to compare two different operative techniques regarding weight loss, resolution of comorbidities and complications. The primary endpoint is weight loss evaluated by %EWL (excessive weight loss, %). The secondary endpoints include resolution of associated comorbidities, improvement of QOL, mortality and morbidity of the procedures.

**Design of the study**

The study is carried out at three centers, Turku University Hospital, Vaasa Central Hospital and Helsinki University Hospital (Peijas). The patients evaluated for enrollment are assigned to undergo surgical treatment for morbid obesity and their treatment follows established treatment protocols.

Inclusion criteria:

- BMI ≥ 40 or BMI ≥ 35 with a significant obesity related comorbidity
- Age 18 – 60 years
- Previous successfully instituted and supervised but failed adequate diet and exercise program

Exclusion criteria:

- BMI > 60
- Significant psychiatric disorder
- Severe eating disorder, active alcohol or substance abuse
- Active gastric ulcer disease
- Difficult GERD with a large hiatal hernia
- Previous bariatric surgery

Preoperative evaluation

All the patients undergo a thorough multidisciplinary evaluation (an endocrinologist, a dietician, and a bariatric surgeon), and a psychiatric evaluation is obtained, if considered necessary. All patients undergo upper gastrointestinal endoscopy and abdominal ultrasound examination. Possible

Helicobacter pylori infection and associated gastric ulcer disease are treated before surgery. Only symptomatic gallstones are considered an indication for laparoscopic cholecystectomy at the time of the bariatric procedure. After the clinical decision of proceeding to bariatric surgery for treating morbid obesity is reached, patient eigibility for this RCT is evaluated according to the inclusion and exclusion criteria. A written informed consent is received and the patients are randomized by a closed envelope method either to undergo laparoscopic sleeve gastrectomy or laparoscopic gastric bypass. Preoperative quality of life is measured by the Moorhead-Ardelt questionnaire at the same visit.

**Postoperative treatment**

Postoperative treatment is carried out according to established treatment protocols. Oral fluids are initiated on the first postoperative day and patients are discharged from the hospital from the second postoperative day onward depending on recovery. All patients are placed on multivitamins postoperatively and proton pump inhibitors are routinely used for the first three months. The postoperative control visits at surgical outpatient clinic are planned at 3, 6, 12, 18 months, 2, 3, 4, 5, 7, 10 and 15 years. At these visits the patients are measured for weight, checked for blood samples according to normal treatment protocols and asked to fill the Moorehead-Ardelt quality of life questionnaire. Plastic surgery consultation is obtained, if needed.

**Addition to 10-year follow-up, ethical amendment 4.2.2018:**

The incidence of gastroesophageal reflux disease (GERD) after bariatric surgery has been under vast research in recent years during the SLEEVEPASS trial follow-up time, especially after sleeve gastrectomy as there have been reports of increased cumulative incidence of Barrett’s esophagus for up to 17% at 10 years after sleeve^18^. The majority of these studies do not have preoperative gastointestinal endoscopy making the evaluation of de novo findings impossible. Obesity itself is also a refluxogenic condition and potential GERD and endoscopic findings need to be assessed after both LSG and LRYGB.In this SLEEVEPASS study, all patients underwent preoperative gastroesophageal endoscopy, and thus the evaluation of de novo findings is possible. With the long-term follow-up endoscopy, we will aquire important information in the actual cumulative incidence or prevalence of Barrett’s esophagus after bariatric surgery. This 10-year follow-up is important not only for individuals (medically essential in light of recent studies), but also globally concidering the major increase in the number of bariatric surgery procedures in the last two decades and this is especially true for LSG, if the Barrett’s incidence results are validated. All available SLEEVEPASS trial patientswill undergo upper gastrointestinal endoscopy at 10 years, and these findings will increase the understanding of the potentially required future postoperative assessments after bariatric surgery even globally. In addition, the patients will fill out the GERD-HRQOL questionnaire to assess subjective GERD symptoms and PPI medication use.

**Current state of the study**

The statement for approval by the ethics committee of Turku University Hospital will be submitted ann the approvals by the ethics committees of Vaasa Central Hospital and Helsinki University Hospital will also be applied. The randomization for the study will be started during spring 2008 after the approvals by the ethics committees. The researchers (Paulina Salminen, Jari Ovaska, Mika Helmiö, Mikael Victorzon, Pekka Tolonen, Pipsa Peromaa, Anne Juuti and Marja Leivonen) will be personally responsible for the preoperative visits, operations and postoperative visits.

**A summary of the addition to the ethical amendment 4.2.2018:**

The 5-year outcomes of the SLEEVEPASS study were published in JAMA in January 2018^19^. The 5-year individual patient data of the SLEEVEPASS trial and a similar Swiss RCT (SM-BOSS study) have been merged aiming to add to the study power of assessing outcomes by doubling the number of randomized patients also adding to the generalizability of the results. Additional information on T2DM was collected. The 7-year outcomes of the SLEEVEPASS trial have been collected and the data is currently being analyzed. The 10-year follow-up including the additional upper gi-endoscopy and a vast clinical evaluation of GERD will be conducted during 2019-2020.

Changes in study group: MD Risto Juusela has replaced the late MD PhD M. Victorzon at Vaasa Central Hospital. In Turku, PhD student Sofia Grönroos, MD, has joined the study group for the long-term outcome assessment of the study.

**Hypothesis and meaning of the study**

The results of this current RCT study enable comparison on the long-term data between the current golden standard of laparoscopic gastric bypass and laparsocopic sleeve gatrectomy. The hypothesis of the study is that as sleeve can be considered less traumatic and easier and faster to perform compared to RYGB, it could become the procedure of choice in treating morbid obesity provided that long-term results of SG were comparable with those of RYGB. The results can directly be applied to patient care.

**References**

1. Obesity: preventing and managing the global epidemic. Report of a WHO consultation. World Health Organ Tech Rep Ser 2000; 894:i-xii, 1-253.

2. [Adult obesity]. Duodecim 2002; 118(10):1075-88.

3. Adams TD, Gress RE, Smith SC, Halverson RC, Simper SC, Rosamond WD, Lamonte MJ, Stroup AM, Hunt SC. Long-term mortality after gastric bypass surgery. N Engl J Med 2007; 357(8):753-61.

4. Balsiger BM, Kennedy FP, Abu-Lebdeh HS, Collazo-Clavell M, Jensen MD, O'Brien T, Hensrud DD, Dinneen SF, Thompson GB, Que FG, Williams DE, Clark MM, Grant JE, Frick MS, Mueller RA, Mai JL, Sarr MG. Prospective evaluation of Roux-en-Y gastric bypass as primary operation for medically complicated obesity. Mayo Clin Proc 2000; 75(7):673-80.

5. Buchwald H, Estok R, Fahrbach K, Banel D, Sledge I. Trends in mortality in bariatric surgery: a systematic review and meta-analysis. Surgery 2007; 142(4):621-32; discussion 632-5.

6. Buchwald H, Williams SE. Bariatric surgery worldwide 2003. Obes Surg 2004; 14(9):1157-64.

7. DeMaria EJ, Sugerman HJ, Kellum JM, Meador JG, Wolfe LG. Results of 281 consecutive total laparoscopic Roux-en-Y gastric bypasses to treat morbid obesity. Ann Surg 2002; 235(5):640-5; discussion 645-7.

8. Higa KD, Ho T, Boone KB. Laparoscopic Roux-en-Y gastric bypass: technique and 3-year follow-up. J Laparoendosc Adv Surg Tech A 2001; 11(6):377-82.

9. Himpens J, Dapri G, Cadiere GB. A prospective randomized study between laparoscopic gastric banding and laparoscopic isolated sleeve gastrectomy: results after 1 and 3 years. Obes Surg 2006; 16(11):1450-6.

10. Lahti-Koski M, Vartiainen E, Mannisto S, Pietinen P. Age, education and occupation as determinants of trends in body mass index in Finland from 1982 to 1997. Int J Obes Relat Metab Disord 2000; 24(12):1669-76.

11. Lalor PF, Tucker ON, Szomstein S, Rosenthal RJ. Complications after laparoscopic sleeve gastrectomy. Surg Obes Relat Dis 2008; 4(1):33-8.

12. Maggard MA, Shugarman LR, Suttorp M, Maglione M, Sugerman HJ, Livingston EH, Nguyen NT, Li Z, Mojica WA, Hilton L, Rhodes S, Morton SC, Shekelle PG. Meta-analysis: surgical treatment of obesity. Ann Intern Med 2005; 142(7):547-59.

13. Mokdad AH, Marks JS, Stroup DF, Gerberding JL. Actual causes of death in the United States, 2000. Jama 2004; 291(10):1238-45.

14. Moon Han S, Kim WW, Oh JH. Results of laparoscopic sleeve gastrectomy (LSG) at 1 year in morbidly obese Korean patients. Obes Surg 2005; 15(10):1469-75.

15. Regan JP, Inabnet WB, Gagner M, Pomp A. Early experience with two-stage laparoscopic Roux-en-Y gastric bypass as an alternative in the super-super obese patient. Obes Surg 2003; 13(6):861-4.

16. Schauer PR, Ikramuddin S, Gourash W, Ramanathan R, Luketich J. Outcomes after laparoscopic Roux-en-Y gastric bypass for morbid obesity. Ann Surg 2000; 232(4):515-29.

17. Sjostrom L, Narbro K, Sjostrom CD, Karason K, Larsson B, Wedel H, Lystig T, Sullivan M, Bouchard C, Carlsson B, Bengtsson C, Dahlgren S, Gummesson A, Jacobson P, Karlsson J, Lindroos AK, Lonroth H, Naslund I, Olbers T, Stenlof K, Torgerson J, Agren G, Carlsson LM. Effects of bariatric surgery on mortality in Swedish obese subjects. N Engl J Med 2007; 357(8):741-52.

18. Felsenreich DM, Kefurt R, Schermann Met al. Reflux, Sleeve Dilation, and Barrett's Esophagus after Laparoscopic Sleeve Gastrectomy: Long-Term Follow-Up. *Obes Surg*. 2017;10.1007/s11695-017-2748-9

19. Salminen P, Helmio M, Ovaska Jet al. Effect of Laparoscopic Sleeve Gastrectomy vs Laparoscopic Roux-en-Y Gastric Bypass on Weight Loss at 5 Years Among Patients With Morbid Obesity: The SLEEVEPASS Randomized Clinical Trial. *JAMA*. 2018;319(3):241-254.

**New references: #18 and #19**
